# FOXO3-MEDIATED PROTECTION AGAINST VASCULAR DEMENTIA RISK IN A CASE-CONTROL STUDY OF ASIAN AMERICAN MEN

**DOI:** 10.1093/geroni/igac059.956

**Published:** 2022-12-20

**Authors:** Philip Davy, Kalpana Kallianpur, Randi Chen, Timothy Donlon, Brian Morris, Richard Allsopp, Bradley Willcox, Kamal Masaki

**Affiliations:** Kuakini Medical Center, Honolulu, Hawaii, United States; Kuakini Medical Center, Honolulu, Hawaii, Honolulu, Hawaii, United States; Kuakini Medical Center, Honolulu, Hawaii, United States; Kuakini Medical Center, Honolulu, Hawaii, United States; Kuakini Medical Center, Honolulu, Hawaii, United States; University of Hawaii, Honolulu, Hawaii, United States; Kuakini Medical Center, Honolulu, Hawaii, United States; Kuakini Medical Center, Honolulu, Hawaii, United States

## Abstract

The longevity-associated allele of FOXO3 is associated with a significant reduction in cardiovascular disease (CVD) risk in older adults. We hypothesized that dementia, which shares several risk factors with CVD, might also be related to FOXO3 genotype, particularly vascular dementia (VaD). Therefore, we utilized the Kuakini Honolulu Heart Program/Honolulu Asia Aging Study dataset to assess the relation of FOXO3 genotype to dementia risk. Preliminary analyses suggested that VaD had the strongest relation to FOXO3 genotype. Therefore, we performed larger, more detailed study of FOXO3 genotype and VaD risk in a 9-year nest case-control study. Chi-Square test was used to assess the association of dementia with FOXO3 genotype in a sample of 1504 Japanese-American male study participants, aged 71-93 years, at the baseline exam. General Linear Model was utilized to compare VaD risk factors between VaD cases and controls. Multivariate Logistic Regression was used to assess the association of VaD with FOXO3 genotype adjusting for birth year, education, APOE4 and other vascular risk factors. We found a significant protective association for carriers of the principal FOXO3 longevity-associated allele (SNP rs2802292 G allele-carriers) against VaD risk (OR = 0.66, 95% CI = 0.44-0.98, p = 0.0388). The protective association retained significance when controlling for common risk factors for VaD in a multivariate model. These data indicate a potential neuroprotective role for the FOXO3 longevity-associated genotype against vascular dementia. This finding merits validation studies in other cohort studies.

